# Recent progress in exosomal non-coding RNAs research related to idiopathic pulmonary fibrosis

**DOI:** 10.3389/fgene.2025.1556495

**Published:** 2025-03-27

**Authors:** Yajing Wei, Mingyang Hong, Huiming Zhu, Feng Li

**Affiliations:** Department of Clinical Laboratory, Affiliated Nantong Hospital of Shanghai University (The Sixth People’s Hospital of Nantong), Nantong, Jiangsu, China

**Keywords:** idiopathic pulmonary fibrosis, exosomes, non-coding RNAs, function, review

## Abstract

Idiopathic Pulmonary Fibrosis (IPF) is a progressive interstitial lung disease characterized by unknown etiology and limited therapeutic options. Recent studies implicate exosomal non-coding RNAs (ncRNAs) as crucial regulators in IPF. These ncRNAs, including long non-coding RNAs (lncRNAs), microRNAs (miRNAs), and circular RNAs (circRNAs), are involved in cellular processes through various mechanisms of selective packaging, intercellular communication, and signaling pathway integration. LncRNAs such as LINC00470 and PVT1 exhibit pro-fibrotic effects, while others like lnc-DC and THRIL show inhibitory roles; some, including UCA1 and MALAT1, demonstrate bidirectional regulation. In miRNAs, pro-fibrotic agents (e.g., miR-486, miR-223) contrast with inhibitory miRNAs (e.g., miR-34a, miR-126), while miR-21 and miR-155 display dual functions. Similarly, circRNAs such as circ_0000479 and circ_0026344 promote fibrosis, whereas circ_0000072 and circ_0000410 act as inhibitors, with certain circRNAs (e.g., circ_002178 and circ_0001246) exhibiting complex regulatory effects. Exosomal ncRNAs modulate key pathways, including TGF-β and Wnt/β-catenin, influencing IPF progression. Despite their potential, challenges remain in exosome isolation, functional characterization of ncRNAs, and clinical translation. Addressing these barriers through innovative research strategies is essential to leverage exosomal ncRNAs in the management and treatment of IPF. This review comprehensively examines the roles of exosomal ncRNAs in IPF, elucidates their mechanisms and pathway interactions, and discusses future perspectives to enhance understanding and therapeutic strategies for this disease.

## Introduction

**FIGURE 1:**
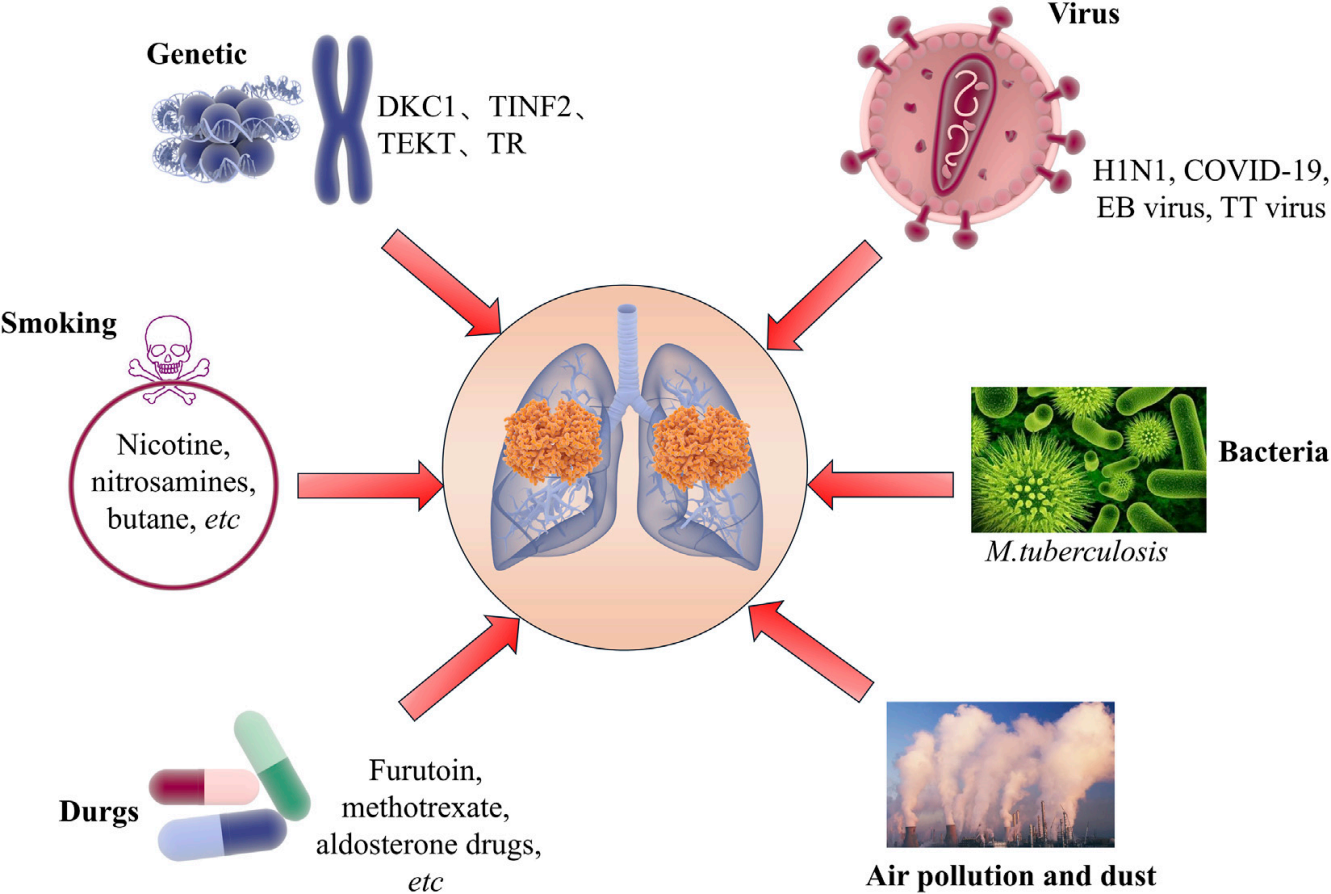
Related pathogenic factors of idiopathic pulmonary fibrosis.

Idiopathic Pulmonary Fibrosis (IPF) is a disease characterized by progressive interstitial lung disease. Its feature is that the lung tissue gradually becomes fibrotic, leading to respiratory dysfunction and eventually respiratory failure (Adkins and Collard, 2012; Lu and El-Hashash, 2019; Varone et al., 2018). The cause of this disease has not yet been clarified, but it is believed that a combination of genetic factors, environmental exposures, and an aberrant wound healing response contributes to the development of IPF (Figure 1). Its clinical manifestations are often similar to those of many other diseases, making early diagnosis somewhat difficult. According to statistical data, the incidence of IPF has been continuously rising worldwide, especially more pronounced among the elderly population (Gulati and Thannickal, 2019; Martinez et al., 2017; Spagnolo et al., 2015). Although in recent years we have gained a deeper understanding of the pathophysiological mechanisms of IPF, the effective treatment methods for this disease are still limited (Collins and Raghu, 2019; Kropski et al., 2013; Shumar et al., 2021). Currently, the main treatment methods include antifibrotic drugs (such as pirfenidone and nintedanib). However, these drugs can only slow down the progression of the disease to a certain extent and cannot cure or reverse the condition. In addition, lung transplantation is the last resort for IPF patients, but due to the scarcity of donor organs and surgical risks, this measure is not applicable to all patients (Balamugesh and Behera, 2007; George et al., 2019; Mason et al., 2007). Therefore, searching for new biomarkers and therapeutic targets has become one of the hotspots in current IPF research.

In recent years, exosomes, as important mediators for cell-cell interaction and information transmission, have received widespread attention (Nguyen et al., 2016). Exosomes are extracellular vesicles with a diameter of approximately 30–150 nm, capable of delivering various biomolecules in the extracellular environment, including proteins, lipids and RNA, etc., (Paul et al., 2023). The formation of exosomes involves the endogenous membrane system and is released into the extracellular space along with the physiological and pathological changes of cells, reflecting the state of the parent cells (Sharma et al., 2017). Studies have shown that exosomes play a crucial role in multiple biological processes such as tumorigenesis, immune regulation and inflammatory responses (Liu Z. et al., 2021). More importantly, non-coding RNAs (ncRNAs) in exosomes, especially microRNAs (miRNAs) and circular RNAs (circRNAs), have significant functions in regulating intercellular communication, the fibrosis process and inflammatory responses (Li C. et al., 2021).

Non-coding RNAs are a class of RNA molecules that do not encode proteins. Broadly speaking, they include microRNAs (miRNAs), long non-coding RNAs (lncRNAs), circular RNAs (circRNAs) and so on (Li et al., 2019; Zang et al., 2021). These RNA molecules play important roles in gene expression regulation, the cell cycle, development and disease progression (Li et al., 2019; Rodrigo, 2018). In recent years, more and more studies have revealed their crucial roles in pathological conditions such as fibrosis (Amalinei et al., 2010; Leitinger, 2014). For example, miRNAs can regulate post-transcriptional gene expression by binding to target mRNAs, while circRNAs regulate the gene expression network by acting as miRNA sponges (Liu Y. et al., 2018; Panda, 2018). Therefore, non-coding RNAs are not only of great significance in basic biological research but also provide new targets for the diagnosis and treatment of diseases (Garcia-Padilla et al., 2018; Gomes et al., 2013).

Studies on Idiopathic Pulmonary Fibrosis (IPF) have shown that non-coding RNAs abundant in exosomes exhibit significant changes in this disease. For example, the expression of some miRNAs in the exosomes of IPF patients is abnormal, which may be closely related to the occurrence and development of pulmonary fibrosis (Njock et al., 2019). Meanwhile, research on circRNAs also shows that they have a potential role in regulating the activation of fibroblasts and the fibrosis process of lung tissue (Wang et al., 2022; Yao et al., 2018). For example, studies have found that miR-143-5p and miR-342-5p in the exosomes of patients with idiopathic pulmonary fibrosis (IPF) inhibit the fatty acid synthesis of alveolar type II cells and promote fibrosis, thereby exacerbating the condition of IPF. Pirfenidone and nintedanib can improve the condition. Another example is that Gan et al.'s study found that three circRNAs (hsa_circ_0044226, hsa_circ_0004099, hsa_circ_0008898) in patients with IPF increased significantly. Moreover, compared with patients with stable IPF (S-IPF), the level of hsa_circ_0044226 in patients with acute exacerbation of IPF (AE-IPF) was significantly higher. In addition, the upregulation of hsa_circ_0044226 was observed in the blood exosomes of a bleomycin-induced IPF mouse model. The expression levels of hsa_circ_0044226, hsa_circ_0004099, and hsa_circ_0008898 in plasma exosomes introduce a new biomarker paradigm for the diagnosis and progression of IPF. By analyzing non-coding RNAs in exosomes, researchers can reveal their potential biomarkers and therapeutic targets in IPF (Chen et al., 2021; Zhang et al., 2021). Therefore, in-depth research on the functions and mechanisms of non-coding RNAs in exosomes will not only help to understand the pathogenesis of IPF but may also provide an important basis for the development of new diagnostic and treatment options.

All in all, as a serious interstitial lung disease, the established pathological mechanism of idiopathic pulmonary fibrosis still requires further research (Strykowski and Adegunsoye, 2023). With the deepening of research on exosomes and the non-coding RNAs they contain, it may provide new ideas and perspectives for the innovation of diagnosis, prognosis evaluation and treatment strategies in the future (Da et al., 2021; Fattahi et al., 2024; Yang et al., 2016). It is hoped that through the exploration of the relationship between exosome non-coding RNAs and IPF, early diagnosis and more effective intervention strategies for this disease can be finally achieved, contributing to the improvement of the quality of life of IPF patients.

In this comprehensive and in-depth review study, we have extensively covered a vast amount of relevant literature. To ensure the comprehensiveness and reliability of the research, we have carefully selected 197 pieces of literature for systematic analysis from numerous documents. These literature come from a wide range of sources, mainly focusing on internationally authoritative databases such as Web of Science, PubMed, and Embase. They cover multiple disciplinary fields closely related to idiopathic pulmonary fibrosis (IPF) and exosomal non-coding RNAs (ncRNAs), such as basic medicine, clinical medicine, as well as cutting-edge disciplines like cell biology and molecular biology. Through a comprehensive analysis of these literature, we are committed to deeply exploring the key information regarding the pathogenesis, regulatory mechanisms, and potential clinical applications of exosomal ncRNAs in IPF, providing a solid theoretical foundation and strong practical guidance for further research and development in this field. The specific article screening method is as follows: Search Strategy: Taking the PubMed database as the main search platform, Boolean logical operators are comprehensively used for the search. The specific search formula is: (“Idiopathic Pulmonary Fibrosis” [MeSH Terms] OR “Idiopathic Pulmonary Fibrosis” [All Fields]) AND (“Exosomes” [MeSH Terms] OR “Exosomes” [All Fields]) AND (“Non - coding RNAs” [MeSH Terms] OR “Non - coding RNAs” [All Fields] OR “lncRNAs” [All Fields] OR “miRNAs” [All Fields] OR “circRNAs” [All Fields]). Specific Keywords: Use “Idiopathic Pulmonary Fibrosis”, “Exosomes”, “Non - coding RNAs”, “lncRNAs”, “miRNAs”, “circRNAs”, etc., As the core search terms, and further expand related vocabulary according to the search results, such as “IPF pathogenesis”, “exosomal ncRNAs function”, “lncRNAs in IPF”, “miRNAs regulation in IPF”, “circRNAs and IPF fibrosis process”, etc., to ensure the comprehensiveness of the search. Inclusion Criteria: The research content focuses on the relationship between exosomal non-coding RNAs and idiopathic pulmonary fibrosis; the literature type is a research paper or a review article; the language is English; the research has a certain degree of scientificity and reliability, with a reasonable experimental design and substantial data. Exclusion Criteria: Literature irrelevant to the research topic, such as those only involving other lung diseases or studies not related to exosomes; literature of poor quality and unreliable data, such as those with too small a sample size or obvious flaws in the research method; duplicate published literature. Publication Time Range: Considering the rapid progress of research in this field, we mainly searched for literature published in the past 10 years (2014–2024) to obtain the latest research results. However, for some classic and important early literature, such as those with pioneering significance in this field published before 2010, they are also retained to ensure the integrity and systematicness of the review.

## Mechanisms of action of exosomal non-coding RNAs

Extracellular vesicles (EVs), especially exosomes, are nano-sized structures bound by membranes (with a size range of 30–150 nm) that are released from different cell types. They play a crucial role as mediators in intercellular communication and carry a wide variety of substances like proteins, lipids, and nucleic acids, among which non-coding RNAs (ncRNAs) are included (Sun et al., 2017; Videira and Da, 2020). Exosomal non-coding RNAs have drawn substantial interest as a result of their multifaceted functions in cellular modulation, disease development, as well as their promising value for therapeutic applications (Alidadi et al., 2023; Fadaei et al., 2022).

### Transport and uptake of exosome ncRNAs

Exosomal ncRNAs can be transported effectively *via* biological fluids like blood, urine, and saliva. This enables them to mediate cell-to-cell communication across relatively long distances (Chen et al., 2022; X; Wang et al., 2024; Wang et al., 2023). This transport takes place through multiple mechanisms, such as passive diffusion, active transport, and specific receptor-mediated endocytosis (Bild et al., 2002; Hastings et al., 2004; Nividha et al., 2024). The lipid bilayer of exosomes shields the RNA they carry from being broken down by ribonucleases outside cells, ensuring its stable transfer to recipient cells. When close to target cells, exosomes can enter them *via* different routes like endocytosis, phagocytosis, or membrane fusion (Kim et al., 2024; 2023; Sabatke et al., 2024). Upon uptake, exosomal ncRNAs are capable of being released into the cytoplasmic compartment, where they commence exerting their regulatory functions. The efficacy of this uptake procedure can be contingent upon numerous factors, such as the specific subtype of recipient cell, the availability of particular receptors on the cell surface, as well as the comprehensive composition of the exosomal membrane (Hushmandi et al., 2024; Willms et al., 2016; Zaborowski et al., 2015). Prior research has emphasized that certain proteins like tetraspanins or integrins present on exosomes can promote their attachment and subsequent uptake by target cells. This notably amplifies the biological influence of the ncRNAs contained within them (Jaquenod et al., 2019).

### Regulatory roles of exosomal ncRNAs

Exosomal non-coding RNAs, such as microRNAs (miRNAs), long non-coding RNAs (lncRNAs), and circular RNAs (circRNAs), are vital in modulating diverse cellular activities. miRNAs, generally short RNA fragments, mainly operate *via* post-transcriptional regulation. They attach to complementary sites on target mRNAs, triggering mRNA breakdown or impeding translation. These mechanisms greatly impact cell proliferation, apoptosis, and differentiation, rendering miRNAs key elements in maintaining normal cellular function and driving disease development (Nasimi et al., 2024; van Wijk et al., 2022). Conversely, long non-coding RNAs exhibit a wide array of regulatory capabilities. They can serve as structural supports for assembling protein complexes, adjust the conformation of chromatin, or even be converted into building blocks for shorter RNAs. Their regulatory scope is not confined to transcriptional control; rather, they’re empowered to coordinate elaborate signaling cascades. Meanwhile, circular RNAs have also become prominent regulators across diverse biological scenarios. Notably, they possess the special function of acting like “miRNA sponges”. By doing so, they block miRNAs from repressing the translation of their target messenger RNAs. Additionally, CircRNAs can interface with RNA-binding proteins to fine-tune gene expression levels.

Exosomal ncRNAs do not just regulate one particular cellular pathway. Instead, they usually manage a complex web of signaling routes, magnifying their impact on how cells work. This broad regulatory power offers promising opportunities to use exosomal ncRNAs in medical treatments. It's especially relevant for diseases where gene expression goes haywire, like cancer and neurodegenerative disorders.

**FIGURE 2:**
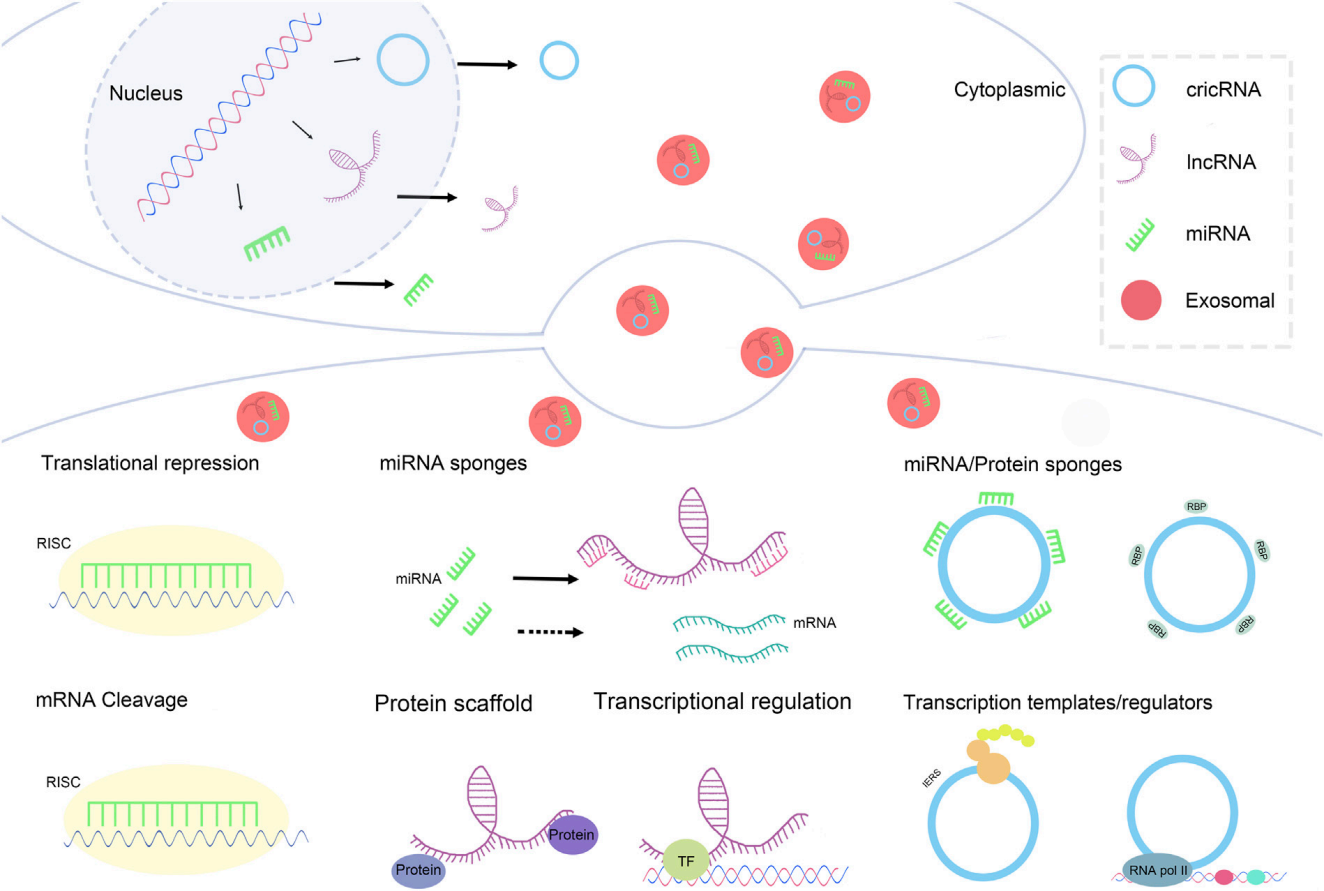
Mechanisms underlying exosomal ncRNAs: a concise overview of pivotal aspects concerning their modes of functioning.

Mechanisms underlying exosomal ncRNAs: a concise overview of pivotal aspects concerning their modes of functioning (Figure 2).

#### Mechanism of action of exosomal miRNA

1) Translational Repression: In the cytoplasm, miRNAs can recognize and bind to the 3′untranslated region (3′UTR) of target mRNAs(Han et al., 2018). This binding recruits the RNA-induced silencing complex (RISC). Once the RISC is associated with the miRNA-mRNA complex, it inhibits the binding of ribosomes to the mRNA (Fukaya and Tomari, 2012), thereby suppressing the translation process. This mechanism effectively reduces protein synthesis without leading to mRNA degradation.
2) mRNA Cleavage: In certain instances, after miRNA binds to RISC, it guides RISC to cleave the target mRNA (M. Zhang et al., 2024). This cleavage primarily depends on the complete or near-complete complementary pairing between miRNA and the target mRNA. The cleaved mRNA fragments are subsequently degraded by cellular nucleases, thus completely blocking the synthesis of the protein encoded by that mRNA.

#### Mechanism of action of exosomal lncRNA

1) Transcriptional Regulation: In the nucleus, lncRNAs can interact with transcription factors (TFs). This interaction can influence gene transcription through various mechanisms (Liu et al., 2018c). For example, lncRNAs can guide transcription factors to specific gene loci to promote the initiation of transcription; alternatively, lncRNAs may alter the activity of transcription factors upon binding, suppressing their association with gene promoters and thereby inhibiting gene transcription (Ferrer and Dimitrova, 2024).
2) miRNA/Protein Sponges: In the cytoplasm, lncRNAs can simultaneously bind to miRNAs and proteins, functioning as miRNA/protein sponges (Dang et al., 2018). By binding miRNAs, they can impact miRNA regulation of target mRNAs; through binding proteins, they can regulate the activity, localization, or stability of those proteins, further influencing various physiological processes within the cell.
3) Protein Scaffold: lncRNAs can also serve as protein scaffolds, facilitating the formation of protein complexes (Ribeiro et al., 2018). They can gather multiple proteins together, allowing these proteins to interact at specific cellular locations and times, thereby forming functional protein complexes that participate in crucial physiological processes such as signal transduction and gene expression regulation.

#### Mechanism of action of exosomal CircRNA

1) miRNA Sponges: In the cytoplasm, CircRNA can function as miRNA sponges (Hoffmann et al., 2023). CircRNA possesses multiple binding sites for miRNAs, allowing it to sequester large amounts of miRNA, thereby reducing the pool of free miRNA. This decrease diminishes the chances of miRNA binding to target mRNAs, indirectly alleviating the inhibitory effect of miRNA on its target mRNAs and subsequently affecting gene expression.
2) Transcription Templates/Regulators: Within the nucleus, CircRNA can act as a transcription template or regulatory factor involved in the regulation of gene expression (Qi et al., 2021). As a transcription template, CircRNA may participate in the synthesis of other RNA molecules; as a regulatory factor, it can influence the initiation and progression of gene transcription through interactions with other transcription-related factors.

To sum up, exosomal ncRNAs embody a new and active means of cell-to-cell communication. By regulating gene expression, they impact diverse biological processes. Grasping how they’re transported, taken up by cells, and how they work mechanistically will speed up the creation of novel treatment methods. It will also deepen our understanding of disease mechanisms, especially in cancer and other illnesses where ncRNA expression patterns are abnormal (Dill and Naya, 2018; Rodriguez et al., 2018).

## The role of exosomal miRNAs in idiopathic pulmonary fibrosis

### Exosomal MiRNAs are involved in the promotion of idiopathic pulmonary fibrosis

In the research on idiopathic pulmonary fibrosis (IPF), microRNAs (miRNAs) in exosomes have been found to be involved in promoting this disease (Elliot et al., 2022). In particular, three miRNAs represented by miR-486, miR-223 and miR-199a have attracted widespread attention in the scientific community. MiR-486 has been shown to be significantly upregulated in the lung tissues and exosomes of IPF patients in multiple studies (Ji et al., 2015). This miRNA can enhance the activation and proliferation of fibroblasts by targeting and inhibiting the expression of certain anti-fibrotic genes. In addition, miR-486 has also been found to promote the deposition of collagen by regulating the synthesis of extracellular matrix components, thus accelerating the fibrosis process of lung tissues (Liu et al., 2019). Its mechanism involves the regulation of cell signaling pathways such as TGF-β and Wnt/β-catenin, driving this complex pathological process. MiR-223 also plays an important role in promoting lung fibrosis (Hou et al., 2023). Studies have shown that the increased expression level of miR-223 is closely related to the severity of IPF (Shan et al., 2015). This miRNA enhances the production of pro-inflammatory cytokines by targeting specific deacetylases and other genes related to the inflammatory response, which in turn triggers the activation and proliferation of fibroblasts. This process not only promotes fibrosis in lung tissues but also leads to the persistence of a chronic inflammatory state, forming a vicious cycle and further aggravating the development of the disease. Furthermore, the role of miR-199a in the fibrosis process has received increasing attention. This miRNA also mediates the progression of IPF through multiple mechanisms. It can not only promote the proliferation of fibroblasts but also enhance the reactivity of lung tissues by inhibiting the expression of certain anti-fibrotic factors, making alveolar epithelial cells unable to be effectively repaired after being damaged, resulting in the formation of fibrosis. Studies also show that miR-199a can further amplify the degree of the fibrosis response by affecting the activity of transforming growth factor-β (TGF-β) and myofibroblasts (Savary et al., 2019). Overall, the important promoting roles played by miR-486, miR-223 and miR-199a in idiopathic pulmonary fibrosis not only provide a new perspective for us to understand the pathogenesis of IPF but also point out the direction for future treatment options. (Table 1)

**TABLE 1:** Exosomal miRNAs are involved in regulating IPF.

MiRNAs	Targets	Functions	References
Inhibition of IPF			
miR-34a	SIRT1	MiR-34a reduces the activity of pulmonary fibroblasts by inhibiting the expression of SIRT1	Jiao et al. (2018)
miR-126	VEGFA	MiR-126 reduces inflammation and cell migration by inhibiting the expression of VEGFA.	Schober et al. (2014)
miR-146a	TNF-α	MiR-146a inhibits the inflammatory response and reduces the activation of fibroblasts by down-regulating the expression of TNF-α	McMillan et al. (2013)
miR-143	KLF4	MiR-143 inhibits the proliferation of fibroblasts and the process of fibrosis by downregulating the expression of KLF4	Davis-Dusenbery et al. (2011)
miR-137	EZH2	MiR-137 inhibits the proliferation and migration of fibroblasts by down-regulating the expression of EZH2	Weng et al. (2022)
Promotion of IPF			
miR-486	FGFR2	MiR-486 promotes the proliferation and activation of fibroblasts by upregulating the expression of FGFR2	(F. Zhang et al., 2024)
miR-223	RhoA	MiR-223 promotes cytoskeletal remodeling and the migration of fibroblasts by inhibiting the expression of RhoA	Vickers et al. (2014)
miR-199a	HIF-1α	MiR-199a enhances the metabolic activity of fibroblasts and stimulates cell proliferation by increasing the expression of HIF-1α, leading to exacerbation of fibrosis	Wang et al. (2019)
Both inhibition and promotion of IPF			
miR-21	PTEN	MiR-21 promotes the proliferation of fibroblasts and inhibits apoptosis by suppressing the expression of PTEN, thereby exacerbating pulmonary fibrosis. On the other hand, miR-21 may also exert antifibrotic effects by regulating specific signaling pathways under certain conditions	Hao and Zhang (2019)
miR-155	SOCS1	MiR-155 can promote inflammatory responses and the activation of fibroblasts, thereby facilitating the progression of fibrosis. However, in an anti-inflammatory environment, it may inhibit fibrosis by reducing inflammatory responses	Wei et al. (2017)
miR-29	COL1A1	MiR-29 overexpression can inhibit collagen synthesis and alleviate fibrosis, while its deficiency promotes collagen deposition, leading to disease progression	Deng et al. (2017)
miR-200	ZEB1	MiR-200 inhibits the expression of ZEB1, promoting the maintenance of epithelial cell phenotypes and reducing fibrosis. However, under certain conditions, it may also facilitate cell transformation, leading to the opposite effect	Bendoraite et al. (2010)

### Exosomal MiRNAs are involved in the inhibition of idiopathic pulmonary fibrosis

In the research on idiopathic pulmonary fibrosis (IPF), microRNAs (miRNAs) in exosomes have been found to play inhibitory roles through multiple mechanisms. In particular, five miRNAs, namely, miR-34a, miR-126, miR-146a, miR-143 and miR-137, are especially important in this regard. As a crucial regulator, miR-34a exerts an inhibitory effect on the proliferation and migration of fibroblasts by targeting multiple profibrotic signaling pathways (Zhang et al., 2012). Studies have found that miR-34a can downregulate the expression of transforming growth factor-β (TGF-β), thereby effectively reducing collagen synthesis and alleviating the fibrosis process, demonstrating its potential in protecting lung tissues (Huang et al., 2018). Moreover, miR-126 also shows a significant inhibitory effect in IPF. Studies indicate that this miRNA enhances vascular integrity by regulating endothelial cell functions and inhibiting inflammatory responses, reducing alveolar damage caused by inflammation and the abnormal proliferation of fibroblasts. The upregulation of miR-126 is believed to help promote the repair process of the lungs and prevent the development of fibrosis (Ohta et al., 2020). Meanwhile, miR-146a plays an important role in the pathogenesis of IPF due to its ability to regulate inflammation. It reduces the excessive activation of immune cells by inhibiting the release of pro-inflammatory factors, thereby alleviating the fibrotic response of lung tissues and showing a good protective effect (Hadjicharalambous and Lindsay, 2018). MiR-143 participates in the inhibition of IPF through different mechanisms (Tam et al., 2011). Relevant studies have pointed out that miR-143 can limit the proliferation of fibroblasts and the deposition of collagen by regulating the cell cycle and metabolic pathways. Its ability to inhibit signaling pathways provides new ideas for the treatment of pulmonary fibrosis. At the same time, miR-137 has also been found to have the potential to inhibit idiopathic pulmonary fibrosis (Lee et al., 2010). It further alleviates the degree of fibrosis by interfering with the proliferation signals of fibroblasts and the expression of inflammation-related factors (Table 1).

In conclusion, these five exosomal miRNAs, namely, miR-34a, miR-126, miR-146a, miR-143 and miR-137, play a key role in the inhibitory mechanism of idiopathic pulmonary fibrosis. They not only reduce the proliferation and migration of fibroblasts by inhibiting the expression of profibrotic factors but also promote the repair and regeneration of lung tissues by regulating inflammatory responses, providing new potential targets for the treatment of IPF. Future research is expected to further explore the specific functions of these miRNAs in IPF and their application prospects as biomarkers and targeted therapies, providing a theoretical basis and practical guidance for the improvement of clinical intervention measures.

### Dual role of exosomal miRNAs in IPF: promotion and inhibition

In the research on idiopathic pulmonary fibrosis (IPF), microRNAs (miRNAs) in exosomes have been found to possess complex dual roles. Among them, four miRNAs, namely, miR-21, miR-155, miR-29 and miR-200, have particularly drawn attention for their promoting and inhibiting effects in this process. MiR-21 has been widely studied and is regarded as a profibrotic factor in IPF. It enhances the proliferation of fibroblasts and the synthesis of collagen by targeting and regulating inhibitory genes (such as PDCD4, TP53, *etc.*), thus promoting the development of fibrosis in lung tissues (Pandit et al., 2011). Meanwhile, miR-21 also shows the potential to inhibit fibrosis in specific microenvironments, especially in the repair process after acute injury (Barwari et al., 2018). It can promote the remodeling of alveolar epithelial cells by regulating the balance between apoptosis and proliferation. Another important miRNA, miR-155, also demonstrates its duality in the pathology of IPF (Su et al., 2017). As a pro-inflammatory mediator, miR-155 promotes the progression of fibrosis by enhancing the inflammatory response and facilitating the activation of immune cells, and it plays a promoting role in the proliferation and migration of fibroblasts (Chi et al., 2019). However, studies have shown that under certain circumstances, miR-155 can also play a certain protective role by regulating the immune microenvironment and inhibiting chronic inflammation, thereby alleviating the progression of fibrosis. Therefore, miR-155 plays a complex role in the pathogenesis of IPF and is worthy of in-depth study. MiR-29 is another miRNA that exhibits dual roles in regulating the fibrosis process (Ge et al., 2011). In terms of promoting fibrosis, the downregulation of miR-29 is believed to lead to the excessive synthesis of collagen and other extracellular matrix components, thereby exacerbating fibrosis (Kwiecinski et al., 2012). However, in the inhibition mechanism of fibrosis, miR-29 can also alleviate the condition by negatively regulating the expression of profibrotic factors (Jing et al., 2018). Therefore, the expression level and environmental state of miR-29 directly affect its role in IPF, reflecting its potential as an important regulatory factor (Matsushima and Ishiyama, 2016). Finally, the miR-200 family of miRNAs also shows duality in IPF. This miRNA family can promote pathological changes by regulating epithelial-mesenchymal transition (EMT) during the fibrosis process. However, studies have also found that when the expression level of miR-200 increases, they can inhibit fibrosis-related signaling pathways, reduce the activity of fibroblasts, and then alleviate fibrosis (Yang et al., 2012). By regulating the expression of transcription factors and signaling molecules, miR-200 can play diverse roles under different physiological and pathological states (Martinez-Campa et al., 2024). In conclusion, exosomal miRNAs such as miR-21, miR-155, miR-29 and miR-200 play complex dual regulatory roles in idiopathic pulmonary fibrosis, which can both promote the occurrence of inflammation and fibrosis and inhibit these pathological processes under appropriate conditions (Table 1).

## The role of exosomal long non-coding RNAs in idiopathic pulmonary fibrosis

### Exosomal LncRNAs are involved in the promotion of idiopathic pulmonary fibrosis

In the research on idiopathic pulmonary fibrosis (IPF), recent findings have shown that exosomal long non-coding RNAs (lncRNAs) play a significant promoting role in the development of this disease. In particular, the studies on two types of lncRNAs, namely, LINC00470 and PVT1, have attracted much attention (Huang et al., 2021; Huang W. et al., 2020). The expression of LINC00470 is significantly upregulated in the lung tissues of patients with idiopathic pulmonary fibrosis. Studies have demonstrated that it promotes the fibrosis process by mediating the activities of lung fibrosis-related cell types, such as fibroblasts and alveolar epithelial cells. Specifically, LINC00470 can enhance the activity of the transforming growth factor-β (TGF-β) signaling pathway, facilitating the proliferation and activation of fibroblasts, which in turn leads to the excessive deposition of extracellular matrix components like collagen, and this process directly drives the fibrosis of lung tissues (Huang W. et al., 2020; Nonaka et al., 2008). PVT1 has also been found to be involved in the regulation of fibrosis (Cao et al., 2020; Tian et al., 2022). Studies have shown that PVT1 can compete with miRNAs for binding, regulate the expression of target genes, activate transcription factors and multiple cell signaling pathways, and then influence the apoptosis and proliferation of alveolar epithelial cells (Ji et al., 2021; Li et al., 2018a; Wang et al., 2024). Further research has revealed that the mechanism by which PVT1 promotes pulmonary fibrosis may be closely related to the regulation of the immune response and the remodeling of the inflammatory microenvironment (Table 2).

**TABLE 2:** Exosomal Long Non-coding RNAs are involved in regulating IPF.

LncRNAs	Targets	Functions	References
Inhibition of IPF			
lnc - DC	IL-6	lnc-DC reduces inflammation by inhibiting the expression of IL-6, thereby slowing the activation and migration of fibroblasts	Geng and Tan (2016)
THRIL	TNF-α	THRIL upregulates the expression of anti-inflammatory factors and inhibits the secretion of TNF-α. This process can alleviate fibrosis-related inflammatory responses and reduce the activation of fibroblasts	Xu et al. (2020b)
GAS5	GLUT1	GAS5 inhibits the expression of GLUT1, reducing cellular energy metabolism and thereby decreasing the proliferation and migration of fibroblasts	Tu et al. (2023)
LINC00460	EZH2	LINC00460 inhibits the activity of EZH2, promoting the regulation of epigenetic modifications and reducing the proliferation and activation of fibroblasts	Lian et al. (2018)
FEZF1-AS1	miR-214	FEZF1-AS1 regulates the expression of miR-214, inhibiting its repressive effects on target proteins, thereby enhancing the antifibrotic capacity of cells	Huang et al. (2020a)
SNHG1	KLF4	SNHG1 inhibits the expression of KLF4, reducing the proliferation and transformation of fibroblasts, thus alleviating the fibrosis process	Penke et al. (2022)
LncRNA-ATB	miR-200	LncRNA-ATB downregulates the expression of miR-200, promoting the upregulation of E-cadherin, which enhances the stability and function of epithelial cells and reduces the occurrence of epithelial-mesenchymal transition (EMT), thereby inhibiting the progression of idiopathic pulmonary fibrosis	Liu et al. (2018a), Li et al. (2018a)
Promotion of IPF			
LINC00470	miR-29	LINC00470 promotes the proliferation of lung fibroblasts and the synthesis of collagen by inhibiting the activity of miR-29, thus playing a promoting role in the development of idiopathic pulmonary fibrosis	Khalil et al. (2015)
PVT1	miR-497-5p	LncRNA-PVT1 activates lung fibroblasts *via* miR-497-5p and is facilitated by FOXM1	Li et al. (2021b)
Both inhibition and promotion of IPF			
UCA1	miR-29	UCA1 inhibits the expression of miR-29, promoting the synthesis of collagen, thereby exacerbating fibroblast activation and the progression of pulmonary fibrosis. However, under certain circumstances, UCA1 may also exert a protective effect by modulating fibrosis-related signaling pathways	Chen et al. (2016)
MALAT1	SRSF1	MALAT1 upregulates the expression of SRSF1, promoting the proliferation and migration of fibroblasts, thus enhancing the degree of pulmonary fibrosis. At the same time, MALAT1 may also counteract fibrosis by inhibiting the activity of specific inducing factors through other mechanisms	Sun et al. (2022)
HOTAIR	EZH2	HOTAIR promotes the proliferation of fibroblasts and enhances pulmonary fibrosis by binding to EZH2 and inhibiting the normal function of epigenetic modifications. However, in certain contexts, HOTAIR has also been found to alleviate fibrosis by downregulating the expression of other fibroblast-activating proteins	Zhang et al. (2014)
TUG1	p21	TUG1 downregulates the expression of p21, promoting cell cycle progression and accelerating the proliferation of fibroblasts, which strongly drives the occurrence of fibrosis. However, TUG1 can also exert an inhibitive effect by regulating the expression of adenosine deaminase, enhancing the apoptosis of fibroblasts	Zhang et al. (2018)
H19	let-7	H19 can promote the development of fibrosis by inhibiting the expression of let-7, enhancing the activation of fibrosis-related genes. However, in certain situations, H19 may also exert an antifibrotic effect by regulating other transcription factors, creating a complex regulatory network	Zhang et al. (2019)
NEAT1	STAT3	NEAT1 promotes the activation and proliferation of fibroblasts by upregulating the activity of STAT3, further exacerbating pulmonary fibrosis. However, NEAT1 can also interact with other regulatory factors to reduce the expression of certain pro-fibrotic factors, demonstrating a dual function	Liu et al. (2022)

Both LINC00470 and PVT1 not only provide clues for understanding the underlying pathogenesis but also pave the way for the early diagnosis of IPF and the development of new therapies, highlighting the importance of the close combination of translational medicine and basic research in clinical applications (Benegas et al., 2022; Nakamura and Shimizu, 2023).

### Exosomal LncRNAs are involved in the inhibition of idiopathic pulmonary fibrosis

In the research related to idiopathic pulmonary fibrosis (IPF), more and more evidence shows that exosomal long non-coding RNAs (lncRNAs) play a crucial role in the inhibitory mechanism of this disease (Kaur et al., 2020; Poulet et al., 2020). In particular, seven lncRNAs, namely, lnc-DC, THRIL, GAS5, LINC00460, FEZF1-AS1, SNHG1 and LncRNA-ATB, have attracted widespread attention. Lnc-DC has been proved in studies to be able to inhibit the occurrence of pulmonary fibrosis by regulating the activation and differentiation of T cells. Specifically, lnc-DC can regulate the expression of anti-inflammatory cytokines and enhance the immune response of T cells, thereby inhibiting the abnormal proliferation of fibroblasts and reducing the formation of fibrosis. The relatively high expression level of THRIL can interact with the expression of tumor necrosis factor-α (TNF-α) and other profibrotic factors, and then inhibit the process of pulmonary fibrosis (Medhat et al., 2022; Sayad et al., 2018). GAS5 exerts its inhibitory effect by inhibiting the transcription of various profibrotic factors and reducing the activation of fibroblasts. Meanwhile, it also affects the regulation of the cell cycle and uses this mechanism to intervene in the fibrosis process of lung tissues (She et al., 2020; Tao et al., 2020). Studies on LINC00460 have shown that its presence in exosomes helps to inhibit the migration and proliferation of fibroblasts. Its mechanism involves regulating multiple key signaling pathways, thus reducing the degree of fibrosis (Yue and Zhang, 2018). Meanwhile, FEZF1-AS1, through its interaction with miRNAs, mediates the inhibition of the inflammatory response and guides the formation of a balance among pro- and anti-fibrotic transcription factors, thereby weakening the progression of idiopathic pulmonary fibrosis (Liu et al., 2024). SNHG1 has also been found to have an inhibitory effect on fibrosis in exosomes. It effectively slows down the development of IPF by downregulating the expression of fibrosis-related genes and interfering with the excessive accumulation of the extracellular matrix (Alvarado-Vasquez et al., 2024; Hewlett et al., 2018). Finally, LncRNA-ATB has been confirmed to have certain potential in the treatment of IPF(Lu et al., 2020; Qi et al., 2017). It plays a positive role in promoting the repair and regeneration of alveolar epithelial cells by regulating the interaction between inflammation and fibrosis, and inhibits the deterioration of fibrosis. In conclusion, these seven exosomal lncRNAs play important roles in the inhibitory mechanism of idiopathic pulmonary fibrosis. Through multiple mechanisms such as interfering with cell signaling pathways, regulating the expression of cytokines and promoting immune functions, they are expected to provide new strategies for the prevention and treatment of IPF (Table 2).

### Complex role of exosomal lncRNAs in modulating IPF: promotion and inhibition

In the research on idiopathic pulmonary fibrosis (IPF), exosomal long non-coding RNAs (lncRNAs) display complex regulatory roles involving both promotion and inhibition. Among them, six lncRNAs, namely, UCA1, MALAT1, HOTAIR, TUG1, H19 and NEAT1, are especially significant. UCA1 can promote the process of pulmonary fibrosis under certain conditions by upregulating the expression of profibrotic factors and activating fibroblast functions, thus accelerating the remodeling of alveolar structures and fibrosis (Li et al., 2021; Yang et al., 2022). However, studies also indicate that UCA1 may have an inhibitory effect in some cellular environments, enabling cells to restore normal functions under stress conditions, reflecting its dual roles in different physiological states (Aalijahan and Ghorbian, 2020). MALAT1 has been widely studied and found to exhibit bidirectional regulatory characteristics at different stages of IPF (Li et al., 2019a). In the early stage of fibrosis development, this lncRNA may promote disease progression and enhance fibroblast proliferation and migration. But after cell injury, MALAT1 may inhibit excessive fibrotic responses by regulating the apoptotic pathway (Huang et al., 2019). HOTAIR also shows a similar duality in IPF. For promotion, it can increase fibroblast activity by activating the Wnt/β-catenin signaling pathway. In terms of inhibition, some studies suggest that HOTAIR may play a catalytic role in lung tissue repair by regulating anti-fibrotic pathways and various cytokines (Hadjicharalambous et al., 2018). TUG1 has a dual function as well. It functions in the profibrotic process by enhancing fibroblast activity (Gu et al., 2019). Meanwhile, under certain conditions, it can inhibit the phosphatidylinositol 3-kinase (PI3K) signaling pathway, thereby reducing the secretion of fibrosis-related cytokines and showing a protective effect. H19 is regarded as playing an important role in understanding the pathology of IPF (Wan et al., 2020). The mechanisms of its simultaneous activation and inhibition are complex and still need further exploration. When cells are in a profibrotic state, H19 can promote fibrosis by increasing collagen synthesis. However, in the repair stage after fibrosis, H19 can inhibit by interfering with the expression of profibrotic factors, helping the regeneration and recovery of alveolar epithelial cells (Tang et al., 2016). Regarding NEAT1, its role in IPF has drawn increasing attention. It may promote the fibrosis and repair processes of cells by regulating the inflammatory response and also show the ability to inhibit the progression of the disease course in some situations, regulating the cell cycle and migration. In summary, these six exosomal lncRNAs are intricately intertwined in the promotion and inhibition mechanisms of idiopathic pulmonary fibrosis. The same lncRNAs exhibit different regulatory effects under different biological backgrounds and cellular states, making them important molecular markers for understanding the pathological process of IPF and providing new ideas for the development of future treatment strategies (Table 2).

## The role of exosomal circRNAs in idiopathic pulmonary fibrosis

### Exosomal CircRNAs are involved in the promotion of idiopathic pulmonary fibrosis

In the research on idiopathic pulmonary fibrosis (IPF), circular RNAs (circRNAs) in exosomes are increasingly recognized as playing important roles in promoting the disease. Multiple studies have shown that circular RNAs such as circ_0000479, circ_0026344, circ_0136662, circRNA_002453, circRNA_001569, circRNA_103011, circRNA_004666 and circFBXW7 are all significantly upregulated in IPF, and their abnormal expressions have been confirmed to be closely related to the activation, proliferation of fibroblasts and the fibrosis process (Surendran et al., 2024). Specifically, circ_0000479 promotes the proliferation of fibroblasts and enhances the expression of multiple profibrotic factors by regulating specific signaling pathways, thus directly promoting the fibrosis process (Wang et al., 2020). Similarly, the upregulation of circ_0026344 is associated with the pathological progression of IPF. Studies have found that it promotes the fibrosis of lung tissues by enhancing the synthesis of extracellular matrix components like collagen (Tjin et al., 2017). Moreover, circ_0136662 is found to enhance the activity of fibroblasts and collagen synthesis by influencing the TGF-βsignaling pathway, providing an additional impetus for fibrosis. Its mechanism may involve acting as a sponge for specific miRNAs, thereby relieving the inhibition on profibrotic factors (Kang, 2017). CircRNA_002453 plays an important role in the processes of inflammation and lung fibrosis (Ouyang et al., 2018). It further aggravates the damage to lung tissues and the development of fibrosis by upregulating the expression of pro-inflammatory factors and enhancing the inflammatory response. Another important circRNA, circRNA_001569, has been shown to promote the formation of lung fibrosis by regulating apoptosis and fibroblast functions (Ling et al., 2024). Based on this, studies on circRNA_103011 have shown that its expression is increased in the exosomes of IPF patients and is closely related to the proliferation and migration of fibroblasts (Yang et al., 2018). This circRNA enhances the survival ability of fibroblasts by influencing the cell cycle and metabolic pathways, thus promoting the progression of the disease. Meanwhile, circRNA_004666 is considered to have the potential to regulate fibroblast activity (Fukuda, 2020). It further promotes the formation of fibrosis by targeting and regulating fibrosis-related genes and increasing the activity in signal transduction pathways. CircFBXW7 shows a dual regulatory role in the processes of cell proliferation and apoptosis (Liu T. et al., 2021). By balancing the growth and death mechanisms of cells, it aggravates the inflammatory and fibrotic responses in IPF (Table 3).

**TABLE 3:** Exosomal circRNAs are involved in regulating IPF.

CircRNAs	Targets	Functions	References
Inhibition of IPF			
circ_0000072	miR-192	Circ_0000072 inhibits the proliferation and migration of fibroblasts by upregulating the expression of miR-192	Putta et al. (2012)
circ_0000410	miR-29c	Circ_0000410 targets miR-29c to increase the expression of collagen-degrading enzymes, thereby inhibiting collagen production and alleviating the degree of fibrosis in lung tissue	Yang et al. (2019)
circ_0018287	SIRT1	By upregulating the activity of SIRT1, circ_0018287 can inhibit the activation and proliferation of fibroblasts, thereby reducing the degree of fibrosis	Zerr et al. (2016)
circRNA_000839	ZEB1	CircRNA_000839 effectively slows down epithelial-mesenchymal transition (EMT) by inhibiting the expression of ZEB1, thereby suppressing the migration and activation of fibroblasts	Ashrafizadeh et al. (2020)
circ_102001	PTEN	Circ_102001 upregulates the expression of PTEN, thereby inhibiting the activation of the PI3K/Akt signaling pathway	Zhu et al. (2013)
circRNA_004161	miR-200	By enhancing the expression of miR-200, circRNA_004161 can suppress the expression of fibrosis-related genes and reduce the activation of fibroblasts	Chen et al. (2020)
circBIRC6	miR-21	CircBIRC6 upregulates the expression of miR-21, inhibiting the migration of fibroblasts, reducing collagen synthesis, and thereby exerting a significant inhibitory effect on idiopathic pulmonary fibrosis	Liu et al. (2010b)
circRNA_006227	AXL	CircRNA_006227 reduces the proliferation and migration of fibroblasts by inhibiting the expression of AXL, thereby alleviating the progression of pulmonary fibrosis	Batchu et al. (2013)
Promotion of IPF			
circ_0000479	miR-423	Circ_0000479 promotes the proliferation and activation of fibroblasts and enhances collagen synthesis by downregulating the expression of miR-423	Zhao et al. (2023)
circ_0026344	miR-29a	Circ_0026344 promotes the migration and survival of fibroblasts by inhibiting miR-29a, thereby enhancing the expression of fibrosis-related genes	Wang et al. (2020)
circ_0136662	miR-145	Circ_0136662 promotes the deposition of extracellular matrix by downregulating the activity of miR-145 in fibroblasts	Que et al. (2022)
circRNA_002453	LRP6	CircRNA_002453 promotes the proliferation and migration of fibroblasts by upregulating the expression of LRP6 and activating the Wnt/β-catenin signaling pathway	Ouyang et al. (2018)
circRNA_001569	SOX9	CircRNA_001569 stimulates the activation and proliferation of fibroblasts by enhancing the expression of SOX9, which increases the expression of fibrosis-related genes	Shen et al. (2019)
circRNA_103011	miR-138	CircRNA_103011 inhibits the expression of miR-138, promoting the upregulation of inflammatory factors such as IL-6, thereby enhancing the activation of fibroblasts	Cheng et al. (2022)
circRNA_004666	PTEN	CircRNA_004666 promotes the proliferation and survival of fibroblasts by downregulating the expression of PTEN and activating the PI3K/Akt signaling pathway	Papa et al. (2014)
circFBXW7	Notch1	CircFBXW7 promotes the activation of fibroblasts by upregulating the expression of Notch1	Buratin et al. (2023)
Both inhibition and promotion of IPF			
circ_002178	miR-145	Circ_002178 promotes the proliferation and activation of fibroblasts by downregulating the expression of miR-145, thereby exacerbating the progression of pulmonary fibrosis. However, under certain circumstances, when circ_002178 cooperates with other regulatory factors, it may inhibit the migration of fibroblasts, demonstrating a protective effect	Yang et al. (2013)
circ_0001246	TLR4	Circ_0001246 enhances the inflammatory response by regulating the TLR4 signaling pathway, thereby promoting the activation and migration of fibroblasts. However, circ_0001246 may also reduce the activation of fibroblasts under conditions that suppress the inflammatory response, resulting in a dual regulatory effect	Li et al. (2019c)
circHIPK3	PTEN	CircHIPK3 promotes the activation of the PI3K/Akt signaling pathway and enhances the proliferation and survival of fibroblasts by inhibiting the expression of PTEN. Conversely, under certain conditions, circHIPK3 can also regulate the PTEN pathway to counteract fibrosis, exerting a certain inhibitory effect	Jiang et al. (2022)
circRNA_0001649	KLF4	CircRNA_0001649 promotes the activation and proliferation of fibroblasts by upregulating the expression of KLF4, thereby increasing the risk of pulmonary fibrosis. At the same time, circRNA_0001649 can also exert a certain inhibitory effect by diminishing the activity of some pro-fibrotic signaling pathways	Chandran et al. (2021)
circ_ITCH	miR-22	Circ_ITCH inhibits certain pro-fibrotic factors by upregulating the expression of miR-22, thereby reducing the proliferation and migration of fibroblasts, and exerting an inhibitory effect in idiopathic pulmonary fibrosis. However, in certain situations, circ_ITCH may also promote fibrosis by negatively regulating specific cell signaling pathways	Kuse et al. (2020)

In conclusion, exosomal circRNAs such as circ_0000479, circ_0026344, circ_0136662, circRNA_002453, circRNA_001569, circRNA_103011, circRNA_004666 and circFBXW7 are not only significantly upregulated in idiopathic pulmonary fibrosis but also jointly promote the fibrosis process of lung tissues through different mechanisms.

### Exosomal CircRNAs are involved in the inhibition of idiopathic pulmonary fibrosis

In the research on idiopathic pulmonary fibrosis (IPF), circular RNAs (circRNAs) in exosomes are gradually showing their important roles in inhibiting the fibrosis process (Gan et al., 2024). Multiple studies have demonstrated that eight circRNAs, including circ_0000072, circ_0000410, circ_0018287, circRNA_000839, circ_102001, circRNA_004161, circBIRC6 and circRNA_006227, have the potential to inhibit fibrosis in IPF. Specifically, circ_0000072 has been found to be able to alleviate the progression of fibrosis in lung tissues by regulating fibroblast functions and inhibiting collagen synthesis (Liu G. et al., 2010). Its main mechanism may involve regulating relevant mitochondrial functions and promoting cellular energy metabolism. Moreover, the upregulation of circ_0000410 has been confirmed to effectively inhibit the proliferation and migration of fibroblasts and slow down the occurrence of fibrosis (He et al., 2018). Its effect may be closely related to the inhibition of the expression of multiple profibrotic factors. In this context, studies on circ_0018287 have shown that it inhibits the synthesis of collagen and other extracellular matrix components by acting as a sponge for specific miRNAs, thereby playing a role in inhibiting the progress of IPF (Zhou et al., 2024). Therefore, circ_0018287 not only regulates the fibrosis process at the molecular level but also alleviates lung tissue damage at the cellular level. CircRNA_000839 plays an important regulatory role in the interaction between inflammation and fibrosis (Zhou et al., 2022). It reduces the impact of fibrosis on lung tissues by downregulating the expression of pro-inflammatory factors and inhibiting the activation of fibrotic cells. Another important circRNA, circ_102001, shows its inhibitory effect when regulating fibrosis-related signaling pathways. It weakens the occurrence of fibrosis by reducing the response of fibroblasts to the pathological environment (Gu et al., 2020). Similarly, the research results of circRNA_004161 show that it has a significant upregulation in IPF patients and plays an inhibitory role by influencing the activity and apoptosis process of fibroblasts (Coward et al., 2010). In addition, circBIRC6 is considered to have an inhibitory effect on the occurrence of fibrosis. It alleviates the degree of fibrosis in lung tissues by regulating cell survival pathways (Zhao et al., 2022), while circRNA_006227 shows a significant protective effect in idiopathic pulmonary fibrosis (Prasse, 2015). Its main mechanism may involve slowing down the activation and migration of fibroblasts by regulating the expression of pro-inflammatory and profibrotic signaling pathways. In conclusion, these eight exosomal circRNAs, namely, circ_0000072, circ_0000410, circ_0018287, circRNA_000839, circ_102001, circRNA_004161, circBIRC6 and circRNA_006227, play important roles in the inhibition process of idiopathic pulmonary fibrosis. They work together through different mechanisms to inhibit the proliferation and migration of fibroblasts and the synthesis of extracellular matrix components such as collagen, thereby alleviating the progression of fibrosis in lung tissues. (Table 3)

### Dual roles of exosomal CircRNAs in IPF: promotion and inhibition

In the research on idiopathic pulmonary fibrosis (IPF), circular RNAs (circRNAs) in exosomes exhibit complex dual roles, having both the ability to promote fibrosis and the potential to inhibit the pathological process. Specific circRNAs such as circ_002178 and circ_0001246 are mainly considered as factors that promote fibrosis. Circ_002178 promotes the synthesis of extracellular matrix components such as collagen by regulating the proliferation and migration of fibroblasts and enhancing their response to profibrotic signals (Munoz-Felix et al., 2014), thus facilitating the progression of fibrosis. However, some studies also suggest that circ_002178 may play a protective role by regulating the expression of certain inhibitory factors and preventing over - activated fibroblasts (Vasse et al., 2021). On the other hand, circ_0001246 promotes the activation and migration of fibroblasts by interfering with factors related to anti - fibrosis pathways, which in turn leads to continuous damage and fibrosis of lung tissue. However, under certain circumstances, circ_0001246 may also be involved in regulating the apoptotic process of cells and inhibiting continuous damage to the lungs (Nho et al., 2022). In contrast, circHIPK3 and circRNA_0001649 show more significant inhibitory effects in this field (Xiao et al., 2021). CircHIPK3 inhibits miRNAs with profibrotic characteristics through a sponge - like function, limiting the activation of fibroblasts, thereby reducing collagen synthesis and inflammatory responses and helping to protect lung tissue from fibrotic damage. However, the expression of circHIPK3 may also be regulated by certain external factors, and then promote cell proliferation under stress conditions, forming a relatively complex regulatory mechanism (Lu and Zhang, 2020). Similarly, circRNA_0001649 mainly inhibits the excessive deposition of extracellular matrix by down - regulating the expression of profibrotic factors, thereby alleviating the progression of pulmonary fibrosis (Lu et al., 2022). But it has also been found that in a specific microenvironment, it may promote the activation of certain signaling pathways, leading to minimal activation of fibroblasts, thus having a certain bidirectional regulatory role. Finally, circ_ITCH shows complexity with its unique dual role in IPF. It not only plays an inhibitory role in fibrosis by regulating cell proliferation, apoptosis and inflammatory responses, but may also promote the activation of fibroblasts and collagen synthesis when specific upstream signaling pathways are activated (Xu et al., 2020a). Therefore, circ_ITCH presents diverse regulatory properties in the pathogenesis of IPF and may have completely different effects in different microenvironments and pathological states. Overall, circ_002178 and circ_0001246 as factors promoting fibrosis are in contrast to the inhibitory effects of circHIPK3, circRNA_0001649 and circ_ITCH. Their interactions jointly shape the complex pathological characteristics of idiopathic pulmonary fibrosis. The understanding of the dual roles of circRNAs can not only enrich our understanding of the pathology of pulmonary fibrosis but also open up new directions for the development of its treatment plans (Table 3).

## Signal pathways associated with idiopathic pulmonary fibrosis

**FIGURE 3:**
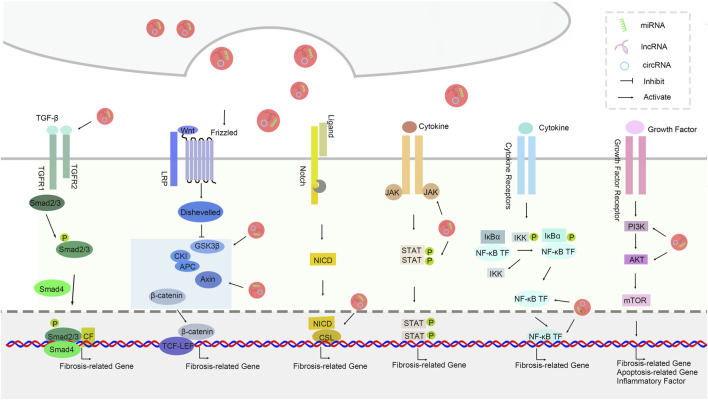
The signal pathways associated with idiopathic pulmonary fibrosis.

The signal pathways associated with idiopathic pulmonary fibrosis are showed below (Figure 3).

### TGF-β signaling pathway

The transforming growth factor-β (TGF-β) signaling pathway plays a central role in the pathogenesis of idiopathic pulmonary fibrosis (IPF). Research indicates that non-coding RNAs in exosomes, especially miR - 21, affect the proliferation and migration of fibroblasts by modulating the TGF-β signaling pathway. miR - 21 can enhance downstream signal transduction by suppressing the expression of the TGF-β inhibitor Smad7, thereby promoting collagen synthesis and deposition. Moreover, the decreased expression of miR - 29 in exosomes also leads to an elevation in TGF-β production, which subsequently triggers alterations in fibrosis-related gene expression, suggesting a close correlation between miR - 29 and the progression of IPF(Guo et al., 2022).

### Wnt/β - catenin signaling pathway

The Wnt/β - catenin signaling pathway also plays a significant role in the process of pulmonary fibrosis. Members of the miR - 135a and miR - 200 family in exosomes have been found to regulate fibrosis-related cell behaviors by inhibiting the activity of β - catenin, a key component of the Wnt signaling pathway. miR - 135a is capable of inhibiting the expression of Wnt target genes, thus impeding the transformation of fibroblasts. The miR - 200 family negatively regulates the epithelial - mesenchymal transition (EMT) process of cells by directly targeting ZEB1, thereby restraining the progression of fibrosis (E. Zhang et al., 2021) (Figure 3).

### PI3K/Akt signaling pathway

The PI3K/Akt signaling pathway is involved in multiple biological processes such as cell proliferation, survival, and apoptosis, and its role in idiopathic pulmonary fibrosis is also garnering increasing attention. Studies have demonstrated that miR - 155 in exosomes promotes the proliferation and activation of fibroblasts by activating the PI3K/Akt signaling pathway, consequently aggravating pulmonary fibrosis. Conversely, the decreased expression of miR - 486 leads to the inhibition of the PI3K/Akt pathway, affecting cell proliferation and survival, further highlighting the crucial role of exosomal non - coding RNAs in regulating this pathway (Sun et al., 2019).

### NF - κB signaling pathway

The nuclear factor κB (NF - κB) signaling pathway regulates inflammatory responses and cell survival and death, and has a substantial impact on the progression of IPF(Jaffar et al., 2021). The upregulation of miR - 146a in exosomes can inhibit the release of inflammatory factors by interfering with the NF - κB signaling pathway, thereby counteracting the formation of pulmonary fibrosis (Luo et al., 2017). Additionally, research has also discovered that long non - coding RNA - p21 (lincRNA - p21) in exosomes has an inhibitory effect on the NF - κB pathway. By interacting with NF - κB, it influences its transcriptional activity, providing a novel perspective for a better understanding of the connection between inflammation and fibrosis.

### Notch signaling pathway

The Notch signaling pathway plays an important role in various physiological and pathological processes, particularly in fibroblast activation. miR - nine in exosomes can indirectly reduce the activation and proliferation of fibroblasts by inhibiting the activation of the Notch signaling pathway. Moreover, studies have shown that miR - 126 inhibits the conversion of endothelial cells to fibroblasts by negatively regulating the Notch signaling pathway. The regulation of the Notch signaling pathway will offer new possibilities for the treatment of IPF, emphasizing the significance of exosomal non - coding RNAs in this signaling network (D. Yang et al., 2022; Zhao et al., 2018).

### JAK/STAT signaling pathway

The JAK/STAT signaling pathway is crucial in cytokine signal transduction and is closely related to the progression of pulmonary fibrosis. Research has found that the miR - 17 family in exosomes can inhibit the activation of this signaling pathway by interfering with the phosphorylation of STAT3, thereby reducing the likelihood of fibroblasts transforming into myofibroblasts. In addition, another important constituent in exosomes, miR - 29b, also modifies the activity of the STAT signaling pathway by suppressing the expression of JAK1, further underlining the potential of exosomal non - coding RNAs in regulating the JAK/STAT pathway (Li et al., 2018b).

In summary, exosomal non-coding RNAs play a vital regulatory role in the signal pathways of idiopathic pulmonary fibrosis. Through different signal pathways, these exosomal RNAs can influence the activation, proliferation, and differentiation of fibroblasts, ultimately affecting the occurrence and development of pulmonary fibrosis. Future research is anticipated to further elucidate the specific mechanisms of these informational molecules in IPF and furnish new targets for the development of novel treatment strategies.

## Discussion

Idiopathic Pulmonary Fibrosis (IPF) is a severe lung disease with a complex pathogenesis and a lack of effective treatment options. The research on exosomal non-coding RNAs (ncRNAs) in IPF has brought new hope for solving this difficult problem. After comprehensively analyzing the various roles of exosomal ncRNAs in IPF, we will further explore their profound significance in the research and treatment of this disease, the numerous challenges they face, and the future research directions.

Exosomal ncRNAs play an essential role in revealing the pathogenesis of IPF. Taking long non-coding RNAs (lncRNAs) as an example, Lnc GAS5 among them can inhibit the transcription of various profibrotic factors and reduce the activation of fibroblasts, thus playing an inhibitory role in the fibrosis process. In the pathological environment of IPF, it can precisely intervene in relevant signal transduction and reduce the excessive deposition of extracellular matrix, providing a key clue for us to deeply understand the molecular regulatory links in the pathogenesis of IPF.

In addition, exosomal ncRNAs show great potential in the clinical application of IPF. Given their relative stability in biological fluids, they are expected to become highly valuable non-invasive biomarkers. For example, the significant changes in the expression levels of some miRNAs in the lung tissue and serum exosomes of IPF patients are closely related to the severity of the disease, which provides a strong basis for the early accurate diagnosis and dynamic monitoring of the disease. Meanwhile, based on a deep understanding of their mechanism of action, exosomal ncRNAs have opened up new targets for the development of innovative treatment strategies. For profibrotic ncRNAs, methods such as using antisense oligonucleotides or RNA interference technology to inhibit their function are expected to reduce the degree of pulmonary fibrosis; for ncRNAs with antifibrotic effects, exploring ways to enhance their expression or activity may become an effective treatment approach for IPF. In addition, exosomes themselves, as natural nanocarriers, have excellent biocompatibility and targeting ability and can precisely deliver therapeutic ncRNAs or other drugs to the lesioned parts of the lungs. By modifying their surface molecules, they can specifically recognize and bind to IPF-related cells, achieving efficient treatment while minimizing side effects on normal tissues, bringing unprecedented opportunities for the treatment of IPF.

However, exosomal ncRNAs face many severe challenges in the research process of IPF. At the technical level, the imperfect exosome isolation and identification techniques have become an important bottleneck restricting the progress of research. The commonly used ultracentrifugation method and immunoprecipitation method each have their drawbacks. The ultracentrifugation method is cumbersome, time-consuming, and prone to damage exosomes; the immunoprecipitation method is costly and may have non-specific binding problems. These factors may all affect the purity and quality of exosome samples, thereby interfering with the accurate detection and functional research of ncRNAs in them. In addition, the lack of unified exosome identification standards also greatly reduces the accuracy and reliability of research results, and it is urgent to establish a unified, standardized, and highly sensitive identification system. In the field of functional research, the function of exosomal ncRNAs is complexly affected by many factors, including the diversity of cell sources, the specificity of target cell types, the dynamic changes of tissue microenvironment, and the progression of disease status. The same ncRNA may have completely different functions in different cell environments or disease stages. For example, miR-21 has both a promoting effect on fibrosis and an inhibitory effect in specific situations in IPF. This complexity and variability of functions greatly increase the difficulty of accurately analyzing its mechanism of action in IPF. At the same time, there is a broad and elaborate interaction network between ncRNAs and other biomolecules (such as proteins and DNA), which further complicates functional research and makes our current understanding of their interaction mechanisms still at a relatively preliminary stage. In the clinical translation stage, the lack of ideal preclinical animal models has become a key obstacle to the translation of exosomal ncRNAs treatment strategies from the laboratory to clinical applications. Although the existing animal models can simulate the pathological characteristics of IPF to a certain extent, there are still significant differences from the pathogenesis and pathological manifestations of human IPF, resulting in the difficulty of directly applying the research results obtained in animal models to clinical practice. In addition, the large-scale production, quality control, and standardization of exosomal ncRNAs as therapeutic drugs are also difficult problems that need to be overcome urgently, including how to ensure the stable yield and high quality of exosomes, how to accurately load ncRNAs and maintain their activity, and how to optimize the drug delivery route, reasonably select the dose, and conduct long-term safety evaluations. All these aspects require in-depth and systematic research.

Looking forward to the future, in order to promote the further development of exosomal ncRNAs in IPF research, we need to continue to work hard in several key directions. In terms of technological innovation, there is an urgent need to develop more advanced, efficient, and highly specific exosome isolation methods. For example, combining microfluidic technology with immunomagnetic bead sorting technology to achieve rapid, high-purity isolation of exosomes while minimizing damage and contamination during sample processing. At the same time, efforts should be made to establish a unified, accurate, and comprehensive exosome identification standard. By comprehensively using cutting-edge technologies such as nanoparticle tracking analysis (NTA), proteomics analysis, and single-cell sequencing, exosomes can be accurately characterized from multiple dimensions, significantly improving the accuracy and reliability of exosome identification. In the field of functional mechanism exploration, fully utilize emerging technologies such as single-cell sequencing and spatial transcriptomics to deeply study the spatio-temporal expression patterns and functional heterogeneity of exosomal ncRNAs in IPF, accurately analyze their specific mechanism of action in different cell types, different pathological stages, and different tissue microenvironments, and comprehensively reveal their dynamic interaction networks with other biomolecules. Through large-scale, multicenter clinical studies, widely collect IPF patient samples, deeply analyze the internal relationship between the expression profiles of exosomal ncRNAs and clinical characteristics and treatment responses, rigorously verify their accuracy and clinical application value as biomarkers, and provide a solid theoretical basis for the personalized and precise treatment of IPF. In the process of promoting clinical translation, actively construct animal models that are more in line with the pathogenesis and pathological characteristics of human IPF. For example, using gene editing technology to construct specific IPF animal models or simulating the complex etiology and pathological process of human IPF through a combination of multiple environmental factors and drug induction to provide a more reliable experimental platform for the preclinical research of exosomal ncRNAs treatment strategies. At the same time, vigorously strengthen the research and development of exosomal ncRNAs therapeutic drugs, optimize the production process and quality control standards, deeply explore suitable drug delivery systems and administration routes, and conduct strict and standardized preclinical safety and effectiveness evaluations to lay a solid foundation for their early clinical application. On this basis, actively and orderly carry out clinical trials, comprehensively evaluate the efficacy and safety of exosomal ncRNAs treatment strategies in IPF patients, and effectively promote their translation from laboratory research to clinical practice, bringing new and effective treatment options for IPF patients and ultimately improving their prognosis and quality of life.
